# Beyond all-or-nothing: why binary thinking undermines harm reduction in addiction medicine

**DOI:** 10.1192/bjo.2025.69

**Published:** 2025-05-15

**Authors:** Luke Manietta, William Drake

**Affiliations:** Southern Illinois University – Carbondale, Carbondale, Illinois, USA; Southern Illinois University School of Medicine, Springfield, Illinois, USA

**Keywords:** Harm reduction, addiction medicine, stigma

## Abstract

In modern healthcare, decision-making favours neatly delineated, categorical imperatives. We prefer to say: ‘This practice is good’ and ‘That one is bad’, believing that each decision has a straightforward yes-or-no resolution. However, medicine thrives in uncertainty, partial improvements and small steps that can lead to life-altering gains. Harm reduction, whether for tobacco use, opioid dependence or beyond, embodies the acceptance of imperfect solutions. It is precisely in these areas that black-or-white thinking can be most destructive. Insisting on total cessation or complete eradication of risk, rather than supporting incremental progress, alienates many patients and perpetuates preventable morbidity and mortality. Recognising this pattern and transcending ‘all-or-nothing’ mindsets is crucial for compassionate, evidence-based care. Accordingly, we ask: ‘How does binary thinking in medical decision-making impact the effectiveness of harm reduction strategies?’ Such an inquiry addresses how well we can truly meet patient needs in real-world practice, especially amid complexity.


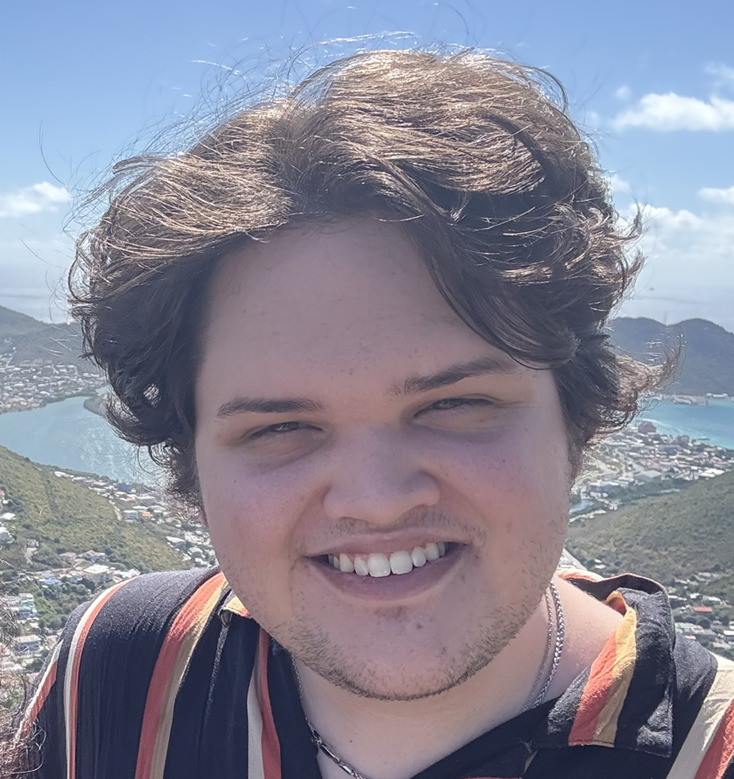


## When ideal solutions fail patients

While harm reduction principles can apply across various medical contexts (e.g. sexual health, palliative care), this discussion primarily focuses on addiction medicine, especially high-risk tobacco use and opioid use disorder. We note that purely medical opioid dependence for chronic pain management is a related but distinct issue, which we reference only briefly to illustrate how binary thinking can similarly pose challenges. From a theoretical standpoint, it is always preferable for a cigarette user to quit entirely or for someone with opioid use disorder to discontinue use, yet real life rarely aligns with such ideals.^1^ Barriers such as deeply ingrained habits, fear of withdrawal, social pressures, mental health struggles and structural inequalities can render a perfect approach either unattainable or unappealing.^2^ Harm reduction evolved precisely for these scenarios, meeting patients where they are in the context of their own life and treatment, as well as acknowledging the difficulty of total abstinence, the continuum of safer choices and the potential for incremental progress.

**Fig. 1:**
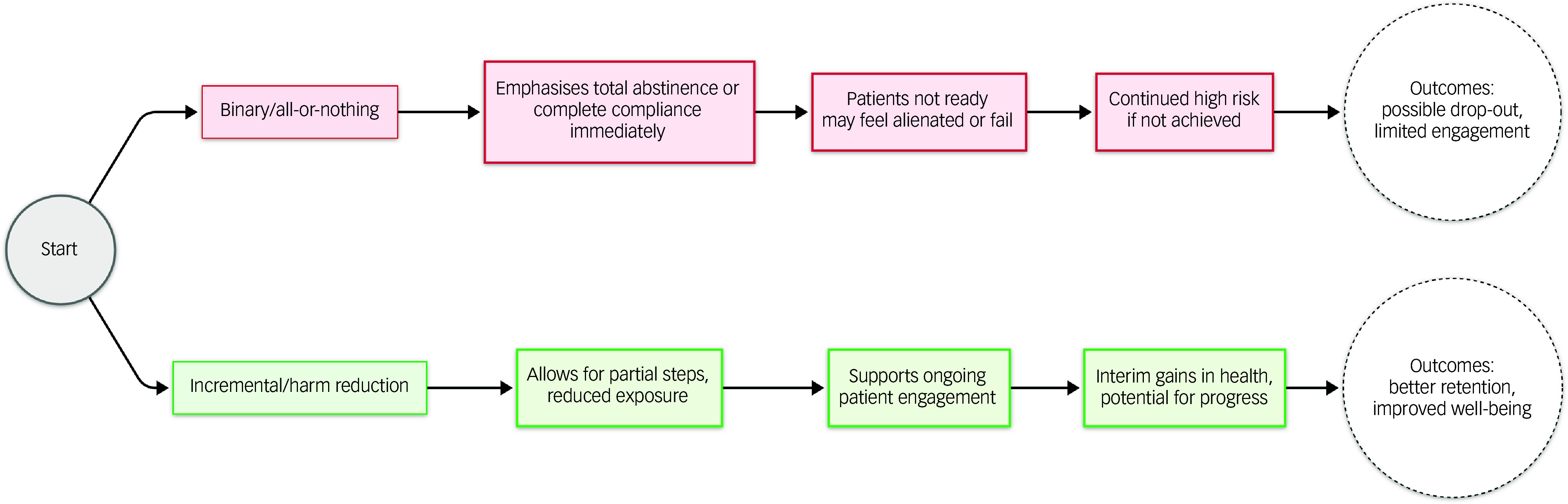
Contrasting ‘binary/all-or-nothing’ versus ‘incremental/harm reduction’ approaches. Patients faced with a strict abstinence requirement often feel alienated when they fail to reach the ideal, whereas a harm reduction strategy fosters engagement and partial progress.

When clinicians or policymakers refuse to adopt anything but the gold standard of total cessation, they tacitly say that rigid abstinence is non-negotiable for a successful outcome. Someone who cannot or will not reach that standard may feel judged, hopeless or even singled out as a lost cause. Denying them incremental or transitional aids, from nicotine replacement and e-cigarettes to fentanyl testing strips, can result in higher levels of harm to individuals and communities.^3,4^ The truth is that not every patient is ready or capable of complete abstinence, and refusing them partial solutions often means leaving them with no risk reduction at all. Ironically, this black-or-white approach can foster the same outcomes (ongoing dependence, higher exposure to toxicants, severe comorbidities) that public health aims to avoid. See Fig. 1 for a conceptual depiction of these contrasting pathways.

## Binary rhetoric in healthcare discourse

Binary rhetoric is common in healthcare for reasons of clarity and risk management. Cognitive heuristics strategies such as anchoring bias (relying on initial impressions) and representativeness heuristic (matching an individual to a disease prototype) favour simplicity over subtleties. It is simpler to prohibit something outright than it is to explain nuanced gradations of risk or to delve into complicated, context-dependent guidelines. In a system already pressed by time constraints, administrative burdens and occasional legal entanglements, giving an all-or-nothing recommendation can feel like the safer option.^5^ This is especially visible in fields such as tobacco cessation counselling and addiction medicine.

The same line of thought emerges in public health campaigns: bold slogans such as ‘No safe level of second-hand smoke’ or ‘Don’t even try it once’ aim to deter harmful behaviours. While these messages can effectively reduce experimentation among non-users, they inadvertently marginalise those already entrenched in the behaviour, making them feel like they must reach an all-or-nothing standard or else be labelled a failure.^6^ Over time, such labelling can drive people away from mainstream healthcare resources.

Binary rhetoric also shapes funding decisions, where agencies frequently channel resources into all-or-nothing approaches rather than multifaceted harm reduction strategies. Such a dichotomy overlooks the reality that not everyone leaps from high-risk behaviour to total abstinence overnight. Many people need phased or assisted transitions. Investment in these bridging strategies can prevent disease, death and further societal costs.^7^

## Consequences of extremism in public perception

When health guidance is overly stark, it can foment distrust in medical advisories. If someone has repeatedly heard that the only healthy approach is total cessation, yet they encounter peers who have improved their health by switching to less harmful products or employing partial protective measures, they may perceive official guidelines as being out of touch. Meanwhile, individuals who are ‘failures’ in an abstinence-only framework might forgo medical follow-up or skip valuable interventions entirely.^8^ They sense that, because they cannot meet the best-case scenario, any lesser effort is meaningless in the eyes of clinicians or public health professionals.

This phenomenon is particularly acute among vulnerable and marginalised populations. People in rural communities who lack resources, individuals experiencing homelessness or those with coexisting mental health disorders can find it nearly impossible to adhere to strict abstinence models.^9^ Harm reduction is designed to help such groups, precisely because it makes room for incremental steps. Imposing black-or-white demands, however well-meaning, can inadvertently exacerbate health disparities.

## Harm reduction as an iterative process

Some in medicine regard harm reduction with unease, often because they view it as a final end-point rather than a stepping stone. For instance, someone might argue that providing e-cigarettes to a smoker who refuses to quit perpetuates nicotine dependence indefinitely; or they stand against harm reduction techniques, arguing that these enable continued participation in risky behaviour. However, in the instance of supervised injection facilities, the health benefits outweigh the risks. In fact, these facilities reduce risk of overdose, improve injection behaviours and increase access to addiction treatment programmes, all without increasing crime and public nuisance.^7^ Nevertheless, many patients who start with moderate harm reduction eventually move on to further improvements – reducing nicotine consumption to zero, stabilising a substance use disorder until they’re prepared for long-term recovery or engaging with counselling that they otherwise might never have considered.^10^ Small positive changes can accumulate into major health advantages over time.

None of this is to dismiss the ultimate value of abstinence for health outcomes. Tobacco is best not used at all, and opioid dependence carries fewer risks when an individual is in sustained recovery. However, these endpoints are not always reachable in a single leap, especially when patients’ psychosocial circumstances or personal motivations are in flux. Harm reduction acknowledges that trajectory matters. Even partial reductions in exposure or modifications in behaviour can have a meaningful, life-saving impact, as often documented in fields such as tobacco control.^4^

Consider a patient who smokes two packs of cigarettes per day. Black-or-white thinking demands that they quit altogether or face contempt for continuing use. Harm reduction might encourage them to transition to a device that eliminates combustion by-products, check for readiness to quit repeatedly and provide ongoing support. Although e-cigarettes are not entirely risk free, the difference in toxicant exposure is substantial and the patient’s sense of autonomy may improve with such an approach.^11^ Rather than alienating them with absolutes, a harm reduction lens fosters a constructive patient–provider relationship that can evolve towards further risk reduction.

## Strategies to overcome binary thinking

Harm reduction is better integrated into public health in the UK than in other parts of the world, namely the USA. This is evidenced by the UK’s adoption of e-cigarettes as a wide-reaching, equitable and low-cost measure to reduce smoking. However, binary thinking is not an issue unique to public health initiatives: it is reinforced by day-to-day clinical activities and nurtured during rigorous medical education. Several principles required by successful harm reduction include humanism, pragmatism, individualism, autonomy, incrementalism and accountability without termination.^12^ Among approaches that can reinforce these principles of harm reduction at an institutional level are the following:

- Ongoing training and education. Providing continuous training for healthcare providers on harm reduction principles and practices is crucial. This includes education on the use of nicotine replacement therapy (NRT), e-cigarettes and fentanyl testing strips. Training should emphasise non-stigmatising care and the benefits of harm reduction strategies.^13^
- Interdisciplinary and team-based care. Establishing interdisciplinary teams that include addiction specialists, primary care providers, mental health professionals and social workers can enhance the implementation of harm reduction strategies. This team-based approach ensures comprehensive care and supports the integration of harm reduction into routine clinical practice.^13^
- Set realistic milestones. Breaking absolute goals into achievable benchmarks (e.g. reducing cigarette consumption by half, switching to a lower-risk product or attending partial counselling sessions) acknowledges that progress need not be all-or-nothing. These smaller milestones can keep patients motivated and connected to the healthcare system.^13^
- Integrate harm reduction into policy. Policymakers should reflect on how blanket prohibitions or stigmatising rhetoric can backfire. Clear guidelines that balance the ideal of abstinence with practical steps towards lowered exposure may help people transition progressively. Specifically, there should be endorsement of safe product alternatives such as e-cigarettes and legislative backing for supervised injection facilities and the distribution of sterilised needles.^14^
- Research intermediate outcomes. Expanding scientific inquiry into partial reductions and scaled improvements can legitimise harm reduction strategies.^15^ If robust evidence shows that halving one’s smoking for 6 months significantly lowers health risks, policy can be updated to encourage partial steps for those who simply are not ready to quit.

## Looking ahead

Medicine advances when it acknowledges complexity, fosters empathy and supports gradual transformation. In truth, refusing to meet patients where they are in their current state of readiness often perpetuates harm more than prevents it. When binary frameworks prevail, physicians restrict their options, health outcomes do not improve and patients lose faith in the system. If public health aims to reduce morbidity and mortality among populations resistant to complete cessation, then it must champion the nuance of harm reduction. Encouraging better choices, rather than the lone perfect choice, empowers patients, fosters trust and can lead to more substantial improvements over time. By moving beyond binary thinking, healthcare providers and policymakers can design interventions that truly respect patients’ situations, progressively minimise risk and uphold the overarching goal of promoting health and well-being across diverse communities.
